# Epigallocatechin-3-Gallate Attenuates Impairment of Learning and Memory in Chronic Unpredictable Mild Stress-Treated Rats by Restoring Hippocampal Autophagic Flux

**DOI:** 10.1371/journal.pone.0112683

**Published:** 2014-11-13

**Authors:** Hong-Feng Gu, Ya-Xiong Nie, Qiao-Zhen Tong, Ya-Ling Tang, Yang Zeng, Kai-Quan Jing, Xi-Long Zheng, Duan-Fang Liao

**Affiliations:** 1 Department of Physiology & Institute of Neuroscience, University of South China, Hengyang, People's Republic of China; 2 Division of Stem Cell Regulation and Application, State Key Laboratory of Chinese Medicine Powder and Medicine Innovation in Hunan, Hunan University of Chinese Medicine, Changsha, People's Republic of China; 3 Department of Neurology of the First Affiliated Hospital, University of South China, Hengyang, People's Republic of China; 4 Smooth Muscle Research Group, Department of Biochemistry & Molecular Biology, Libin Cardiovascular Institute of Alberta, Faculty of Medicine, University of Calgary, Calgary, Alberta, Canada; university of alabama at birmingham, United States of America

## Abstract

Epigallocatechin gallate (EGCG) is a major polyphenol in green tea with beneficial effects on the impairment in learning and memory. Autophagy is a cellular process that protects neurons from stressful conditions. The present study was designed to investigate whether EGCG can rescue chronic unpredictable mild stress (CUMS)-induced cognitive impairment in rats and whether its protective effect involves improvement of autophagic flux. As expected, our results showed that CUMS significantly impaired memory performance and inhibited autophagic flux as indicated by elevated LC3-II and p62 protein levels. At the same time, we observed an increased neuronal loss and activated mammalian target of rapamycin (mTOR)/p70 ribosomal protein S6 kinase (p70S6k) signaling in the CA1 regions. Interestingly, chronic treatment with EGCG (25 mg/kg, i.p.) significantly improved those behavioral alterations, attenuated histopathological abnormalities in hippocampal CA1 regions, reduced amyloid beta1–42 (Aβ_1−42_) levels, and restored autophagic flux. However, blocking autophagic flux with chloroquine, an inhibitor of autophagic flux, reversed these effects of EGCG. Taken together, these findings suggest that the impaired autophagy in CA1 regions of CUMS rats may contribute to learning and memory impairment. Therefore, we conclude that EGCG attenuation of CUMS-induced learning and memory impairment may be through rescuing autophagic flux.

## Introduction

Accumulating evidence suggests that chronic unpredictable mild stress (CUMS) is a significant etiological factor for neurodegenerative disorders characterized by amyloid-β (Aβ) deposition [1], neuron loss [2], learning and memory deficits [3], [4]. CUMS increases corticosterone secretion, which causes dysregulation of hypothalamic–pituitary–adrenocortical (HPA) axis and impairment of hippocampus-dependent learning and memory processes. Clinical data indicate that stress disorders and Alzheimer’s disease (AD) are characterized by HPA dysfunction, and psychological stress increases AD risk [5]. Moreover, in humans and animals, the offspring of mothers that experience stress during pregnancy have been reported to display cognitive dysfunctions [6]. These findings suggest that chronic stress plays a critical role in the development of learning and memory impairment.

The etiological role of dysregulated autophagy in cognitive dysfunctions has been a subject of intense investigation. However, the majority of present studies examining stress contributions to learning and memory deficits have focused on HPA dysfunction, tau phosphorylation and Aβ aggregation in AD transgenic mice [1], [2], [4], and only few studies have focused on the role of autophagy in stress-induced memory impairment. The macroautophagy pathway (hereafter referred to as autophagy) is the main degradation route for damaged organelles [7] and protein aggregates [8], [9]. Autophagy is a highly regulated process characterized by the formation of double or multi­membrane vesicles (autophagosomes) that sequester portions of cytosol. Autophagosome then fuses with a lysosome to form an autolysosome where the captured material is degraded together with the inner membrane. Above dynamic process of autophagy is termed autophagic flux [10], [11]. When autophagic flux is impaired, the subsequent accumulation of damaged organelles and protein aggregates may impair cellular functions and lead to neuronal cells death [12], [13]. These data suggest that autophagy may be intimately associated with stress-induced cognitive dysfunctions. Thus, we investigated whether autophagy mechanism was involved in learning and memory impairment in experimental model of CUMS rats.

In neurons, autophagy acts predominantly as a pro-survival pathway to protect the cells from stress [12], [14], [15]. Autophagic flux impairment is often associated with a number of diseases [8], [16], such as some forms of cancers and neurodegenerative disorders. Several studies have identified specific defects in the autophagy process in AD mouse model [7], [17], [18]. In fact, the strategies to restore autophagic flux were reported to prevent neuropathological and cognitive deficits in the AD mouse model [19]–[21]. These data indicate that activation of autophagy may be a therapeutic strategy in learning and memory deficits.

Epigallocatechin gallate (EGCG), a natural anti-oxidant flavonoid, has a variety of beneficial therapeutic roles, such as anti-inflammatory and neuroprotective effects [22], [23]. We and others have shown that EGCG has beneficial health effects in various pathophysiological conditions including neuronal cells injury [24] and AD-related cognitive deficits [25]. Furthermore, recent research has uncovered an important role for EGCG regulation of autophagic flux in reducing intracellular lipid accumulation in hepatic [26] and vascular endothelial cells [27]. However, it remains largely unknown whether EGCG protects against CUMS-induced memory impairment in rats through its regulation of autophagic flux.

In this study, we investigated the effects of EGCG treatment on the spatial learning and memory functions, pathological changes, and hippocampal autophagic flux in CUMS rats. Here, we found that EGCG treatment could significantly alleviate CUMS-induced memory impairment in rats likely through restoring autophagic flux. Hence, pharmacological manipulation of autophagic flux by EGCG treatment may offer an alternative therapeutic approach in cognitive dysfunctions.

## Materials and Methods

### Reagents

Epigallocatechin-3-gallate (EGCG) (purity >98%, Shanghai, China) was kindly provided by Shanghai U-sea Biotech Co., Ltd. Antibodies against GAPDH, LC3, p62, p-mTOR (Ser^2448^) and p-p70S6K (Thr^389^) were purchased from Abcam (Cambridge, UK). Chloroquine (CQ) was purchased from Sigma (Sigma, USA).

### Animals

60 male Wistar rats were obtained from Peking University Health Science Center (15 weeks, 200–230 g). Animals were maintained on a 12-h light/dark cycle (lights on at 7∶00 a.m., lights off at 7∶00 p.m.) under controlled temperature (23±1°C) and humidity (50±10%), and were given standard diet and water freely. They were allowed to acclimatize for a week before the onset of the experiment. The experiments on animals have been approved by the Animal Experimentation Ethics Committee of the University of South China and conformed to the guidelines of the “China Council on Animal Care”.

### Design and drug treatment

Rats were randomly divided into 6 groups with 10 animals in each: control (non-stressed) group, CUMS group, CQ treatment followed by CUMS (CUMS + CQ); EGCG treatment followed by CUMS (CUMS + EGCG) group, co-administration with EGCG and CQ followed by CUMS (CUMS + EGCG + CQ), and vehicle treatment followed by CUMS (vehicle + CUMS) group. EGCG was dissolved in sterile saline. Rats received 28 injections of EGCG by intraperitoneally (i.p.) in 1 ml volume at a dose of 25 mg/kg, once daily. CQ was injected into the right lateral ventricle of the rat with a constant infusion technique. Briefly, the rats were stereotaxically implanted with the cannulas leading into the right lateral ventricle at coordinates of 0.8 mm anterior to posterior bregma, 1.5 mm mid to lateral, 4 mm dorsal to ventral. The rats received daily intraventricular injections of 5 µl chloroquine diphosphate salt (20 µM) for 28 days. The doses of EGCG and CQ were selected based on previous studies [23], [28]. The drugs (EGCG and CQ) were given 30 min before the stress exposure.

### CUMS protocol

The stress scheme was slightly modified from previous study [29]. Briefly, rats in CUMS groups were exposed to different stressors, namely, 24-h food deprivation, 24-h water deprivation, 5-min cold swimming (at 6°C), 1-min tail pinch (1 cm from the end of the tail), physically restraint for 2 h, exposure to rat odor (removal of the cage containing the experimental rats from the procedure room and placing the experimental rats into cages in which cats had been held) for 1 h and overnight illumination. One of these stressors (in random order) was given every day for 4 weeks. The control rats were housed under identical conditions in a separate room and had no contact with the stressed animals.

### Assessment of plasma corticosterone concentration

After behavioral tests, rats were sacrificed and blood samples were obtained by heart puncture. Samples were centrifuged at 4°C for 30 min at 2500 rpm. The plasma corticosterone concentration was determined by solid-phase^125^I radioimmunoassay using a commercially available reagent kit (Diagnostic Products, Los Angeles, CA).

### Morris water maze assay

In the last week of treatment, spatial memory was assessed by the Morris water maze (MWM) test. The task was conducted in a circular tank (1.25 m diameter and 0.4 m high) filled with opaque water (depth 0.3 m, 25°C). An 8-cm-diameter platform was placed at the center of northwest quadrant of the tank. On the first day, all rats were trained to remember the visible platform. During the following 5 d, the hidden platform trials were used to evaluate spatial learning ability. On each day, the rats were subjected to three trials with 10 min interval between trials. Each trial lasted for 90 s unless the rats reached the platform first. If the rats failed to escape within 90 s, they were gently conducted to the platform and allowed to stay there for 20 s. Escape latency (s) was recorded by video-tracking system. On the 6th day, the platform was removed from the tank and each rat was allowed one 90 s swim probe trial. Finally, the data for the escape latency, the number of platform location crosses and percentage of time spent in the target quadrant between groups were analyzed.

### Histological analysis by hematoxylin and eosin (HE) staining

The rats were given an overdose of pentobarbital (100 mg/kg) and perfused transcardially with phosphate buffer solution (PBS), followed by 4% paraformaldehyde. Brains were removed and stored in 10% formalin for 24 h, followed by 10% formalin with 30% sucrose. After being dehydrated, the brain was embedded in paraffins. Consecutive coronal paraffin sections (4 µm) were collected throughout the hippocampus according to the rat stereotaxic atlas and stained with HE. The number of cells in the hippocampal CA1 region was counted under a light microscope (400× magnification) using the following formula: number of cells per mm region of CA1 =  the number of normal CA1 cells/CA1 length. Cells that underwent morphological changes, such as pyknosis or nuclear fragmentation were excluded.

### Terminal deoxynucleotidyl transferase-mediated dUTP nick end-labeling (TUNEL) assay

Dewaxed sections of rats of each group were washed three times (5 min each) in 0.01 M PBS, and permeabilized in proteinase K for 10 min. Endogenous peroxidase was deactivated by 0.3% hydrogen peroxide. These sections were washed 3 times again. Then they were incubated with TdT at 37°C for 1 h, and incubated with antibody at 37°C for 1 h. These sections were stained by 3, 30-diaminobenzidine (DAB). After hematoxylin post-staining, they were mounted and observed under light microscope. 5 slides were randomly selected from each group, and in each slide, 5 visual fields in the hippocampus were randomly selected. The number of TUNEL-positive cells was counted with about 500 cells counted per slide. The TUNEL-positive cells rate was calculated to equal (the number of TUNEL-positive cells/total cells)×100%.

### Ultrastructural morphological analysis using transmission electron microscope (TEM)

Under anesthesia, 4 rats per group were transcardially perfused with 4% paraformaldehyde and 2.5% glutaraldehyde in PBS. The brains were removed and immediately immersed in 2.5% glutaraldehyde in 0.1 M PBS, pH 7.4 on ice, with shaking for 6 h. After hippocampal CA1 region dissection, fixation was continued for 1 h at 4°C. The material was washed and post-fixed in a 1% O_S_O_4_ in 0.1 M PBS for 30 min and was processed for Epon embedding and stained with lead citrate and uranyl acetate. Ultrathin sections were cut on a Reichert 2 microtome and a minimum of 10 sections from each hippocampus were observed under a transmission electron microscope (JEM-1200EX; JEOL, Tokyo, Japan). Quantification of autophagosomes was performed as previously described [17]. Pictures were taken from randomly selected cells from each sample. For each picture, the number of autophagosomes and the total cellular area were determined and the number of autophagic structures per cell cytoplasmic area was calculated.

### Western blotting analysis

Hippocampal CA1 tissues were treated with CelLytic^MT^ Mammalian Lysis Reagent (Sigma), 1% proteinase K inhibitor, 0.1% Triton X-100 or RIPA buffer that contains NaCl 0.5 M, Tris 50 mM, EDTA-Na 1 mM, sodium dodecyl sulfate (SDS) 0.05%, Triton X-100 0.5%, and phenylme-thanesulphonylfluoride (PMSF) 1 mM. The lysates were centrifuged at 16,000 g, 4°C for 30 min, and the supernatant was stored at −80°C. Protein samples (30 µg) were run on 8–15% SDS-polyacrylamide gel electrophoresis and then transferred onto a nitrocellulose membrane at 90 V for 120 min. Blots were probed with a monoclonal antibody against GAPDH (1∶1000), LC3 (1∶1000), p62 (1∶1000), p-mTOR (1∶1000), and p-p70S6K (1∶1000) at room temperature for 2 h. After primary antibody incubation, the membranes were washed and incubated with horseradish peroxidase-conjugated secondary IgG (1∶3000) for 1 h at room temperature. The immunoreaction was visualized using Amersham Enhanced Chemiluminescence (Amersham Pharmacia Bio-tech, Piscataway, NJ, USA). The densities of blots were analyzed using a scanning densitometer that was operated by Scanner Control software (Molecular Dynamics, Sunnyvale, CA, USA). Results were obtained by calculating the density using Imagequant software (American Biosciences, Pittsburg, PA, USA) and reported as relative optical density of the specific proteins.

### ELISA assay

After the behavioral test, rats were sacrificed, and the brains were removed. The isolated CA1 regions (n = 4 for each group) were weighed and homogenized in 10 volumes of guanidine-Tris buffer (5 M guanidine HCl/50 mM Tris-HCl, pH 8.0). The homogenates were mixed for 3 h in room temperature and centrifuged with 15,000 rpm for 20 min at 4°C. Levels of soluble and insoluble Aβ_1−42_ in the hippocampus were determined by the Aβ_1−42_ ELISA kits (Invitrogen, Camarillo, CA, USA) according to the manufacturer’s instructions. A spectrophotometer was used to read the absorbance of the plates at 450 nm.

### Statistical analysis

All data were presented as mean ± SEM. Student's t-test or one-way ANOVA followed by Newman–Keuls post-hoc test (PRISM software) was used. In those tests, significance level was set at *P*<0.05.

## Results

### EGCG does not reduce the plasma corticosterone levels in CUMS rats

**Figure 1:**
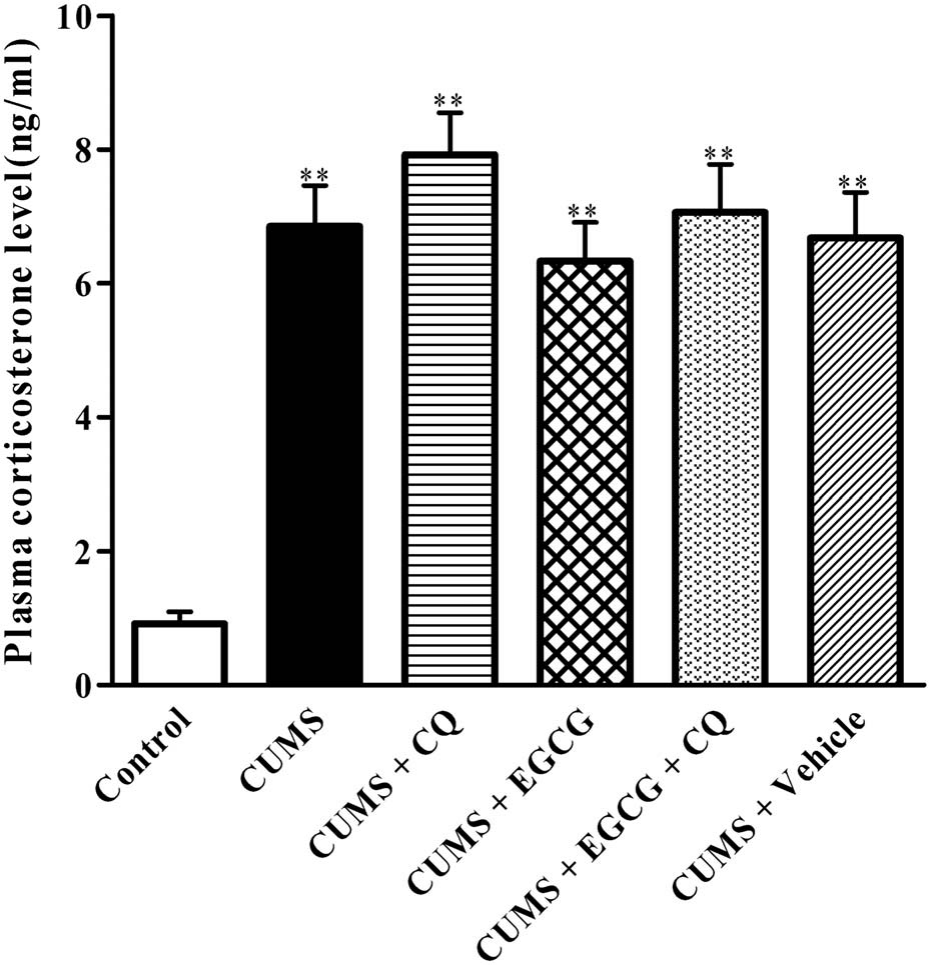
Effects of EGCG on the plasma corticosterone levels in CUMS rats. The plasma corticosterone concentrations were assayed using a radioimmunoassay as described in Methods. Control, control group; CUMS, chronic unpredictable mild stress group; CUMS + CQ, CQ administration followed by CUMS group; CUMS + EGCG, EGCG treatment followed by CUMS group; CUMS + EGCG + CQ, co-administration with EGCG and CQ followed by CUMS group; Vehicle, vehicle treatment followed by CUMS group. The drugs (EGCG and CQ) were given to rats 30 min before the stress exposure. Data are shown as mean ± SEM (n  = 10 for each group). **P<0.01 versus the control group.

To ensure that CUMS paradigms induced sufficient stress, plasma corticosterone concentrations were determined as a means of quantifying stress. As shown in Fig. 1, plasma corticosterone levels increased significantly in the rats exposed to CUMS, as compared with those in the no stressed control ones (*P*<0.01). Plasma corticosterone levels in CUMS + CQ group, CUMS + EGCG group, and CUMS + EGCG + CQ group rats had no significant difference as compared with those in CUMS group rats (*P*>0.05), respectively.

### EGCG markedly ameliorates CUMS-induced learning and memory impairment of rats

**Figure 2:**
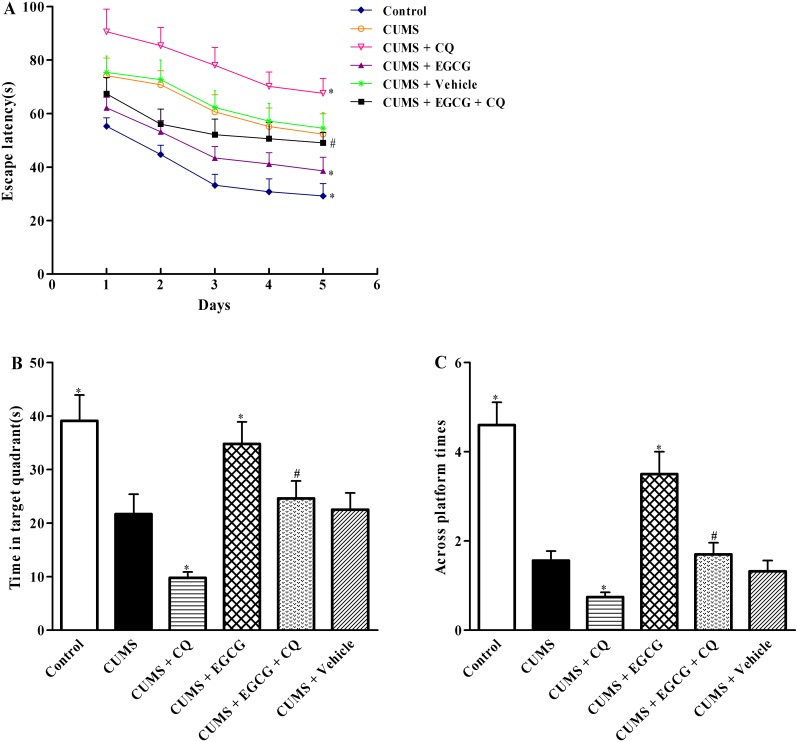
EGCG ameliorates learning and memory deficits in CUMS rats. MWM test was performed to evaluate spatial memory in rats. (A) In 5 d training trials, the escape latencies were measured to assess the rat memory ability. (B, C) In the probe trial, the time spent in target quadrant and the time for rats to cross the area where the submerged platform was placed in training trails were analyzed. Control, control group; CUMS, chronic unpredictable mild stress group; CUMS + CQ, CQ administration followed by CUMS group; CUMS + EGCG, EGCG treatment followed by CUMS group; CUMS + EGCG + CQ, co-administration with EGCG and CQ followed by CUMS group; Vehicle, vehicle treatment followed by CUMS group. The drugs (EGCG and CQ) were given to rats 30 min before the stress exposure. Data are shown as mean ± SEM (n = 10 for each group). *P<0.05 versus CUMS group, ^#^ *P*<0.05 versus CUMS + EGCG group.

First, we performed the MWM assay to examine the effect of EGCG on CUMS-induced memory impairment. The change in the escape latency time to reach the hidden platform was observed in this trial. As shown in Fig. 2A, although there was a downward trend in escape latency time in water-maze training session for 5 d, the mean latency (days 23–27) was significantly prolonged in the CUMS group as compared with the non-stressed control group (*P*<0.05), indicating a poorer learning. Compared with rats in CUMS group, the mean latency of rats in CUMS + CQ group obviously prolonged, suggesting CQ potentiated neuronal dysfunction in CA1 regions induced by CUMS. EGCG treatment, at a daily dose of 25 mg/kg for 28 d, significantly shortened escape latency time as compared with that in CUMS group (*P*<0.05).

Next, platform was removed on day 28 to evaluate the retention of memory. As shown in Fig. 2B, C, rats in CUMS group spent significantly less time in the target quadrant (Fig. 2B) and showed a decreased number of across the target (Fig. 2C) than the control group rats. Average spent time in the target quadrant and number of across the target in CUMS + CQ group remarkably reduced as compared with those in CUMS group, respectively (*P*<0.05). Rats in CUMS + EGCG group exhibited a markedly increased the average time spent in the target quadrant (Fig. 2B) and number of across the target quadrant (Fig. 2C) as compared with those in CUMS group, indicating improvement in cognitive performance. However, CQ, an autophagic flux inhibitor, could abrogated EGCG protective effects against CUMS-induced learning and memory impairment (*P*<0.05), suggesting that autophagic flux may be involved in EGCG improvement of CUMS-induced memory impairment.

### EGCG attenuates CUMS-induced neuronal damage in CA1 region of hippocampus

**Figure 3:**
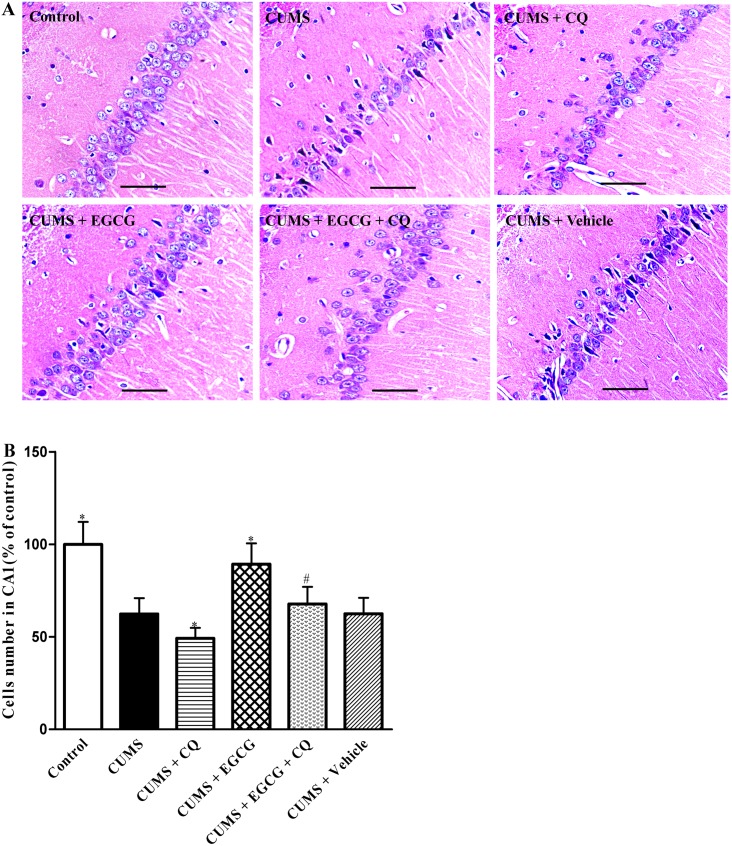
EGCG decreases CUMS-induced cell loss in CA1 region of hippocampus. (A) Representative light micrographs of HE-stained CA1 hippocampal cells. (B) Statistical analyses for the number of cells in the CA1 region. Control, control group; CUMS, chronic unpredictable mild stress group; CUMS + CQ, CQ administration followed by CUMS group; CUMS + EGCG, EGCG treatment followed by CUMS group; CUMS + EGCG + CQ, co-administration with EGCG and CQ followed by CUMS group; Vehicle, vehicle treatment followed by CUMS group. The drugs (EGCG and CQ) were given to rats 30 min before the stress exposure. Data are shown as mean ± SEM (n = 4 for each group). *P<0.05 versus CUMS group, ^#^ *P*<0.05 versus CUMS + EGCG group. Scale bar, 100 µm.

Considering the principal role of hippocampal CA1 region in the learning and memory, we examined the number and arrangement of the CA1 cells by HE staining. As shown in Fig. 3A, the CA1 hippocampal cells in the control group exhibited a regular arrangement with distinct edges and a clear nucleus and nucleolus, and no cell loss was found. In the CUMS group, however, the CA1 cells were irregularly arranged. In many of these cells, the edge, nucleus and nucleolus became ambiguous. For the number of cells in the CA1 area (Fig. 3B), Post-hoc comparisons indicated that the CUMS group showed a significantly smaller number of CA1 cells compared with that in the control group (*P*<0.05). The average number of cells in CQ + CUMS group was lower than that in CUMS group (*P*<0.05). Conversely, these CUMS-induced abnormalities were significantly improved by EGCG treatment. The cells in CUMS + EGCG group had better cell morphology and were more numerous than those in the CUMS group (Fig. 3A, B). However, compared with CUMS + EGCG group, the arrangement (Fig. 3A) of CA1 cells became irregular and cell loss (Fig. 3B) was obviously increased in CUMS + EGCG + CQ group, indicating co-administration of CQ with EGCG markedly reduced the protective effects of EGCG treatment alone. These results suggested that EGCG decreased CUMS-induced neuron loss in CA1 areas of hippocampus via restoring autophagic flux.

### EGCG decreases CUMS-induced apoptotic cells in the CA1 region

**Figure 4:**
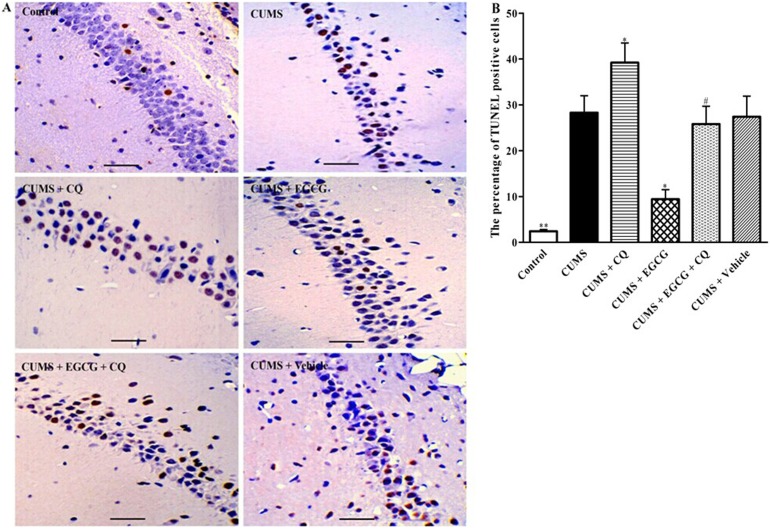
EGCG reduces the number of apoptotic cells in the CA1 region. (A) The TUNEL assay was used to detect apoptotic cells in the CA1 region of rats as described in Methods. (B) Quantification of TUNEL-positive cells. Control, control group; CUMS, chronic unpredictable mild stress group; CUMS + CQ, CQ administration followed by CUMS group; CUMS + EGCG, EGCG treatment followed by CUMS group; CUMS + EGCG + CQ, co-administration with EGCG and CQ followed by CUMS group; Vehicle, vehicle treatment followed by CUMS group. The drugs (EGCG and CQ) were given to rats 30 min before the stress exposure. Data are shown as mean ± SEM (n = 4 for each group). **P<0.01, *P<0.05 versus CUMS group, ^#^ *P*<0.05 versus CUMS + EGCG group. Scale bar, 100 µm.

After characterization of cell number, apoptotic cells in the hippocampal CA1 region of rat were detected by TUNEL method (Fig. 4A, B). The TUNEL-positive cells were rarely detected in the CA1 region of the control group. In contrast, the total number of TUNEL-positive cells was obviously increased in the CUMS rats (Fig. 4A). Percentage of TUNEL-positive cells in CUMS + CQ group was much higher than that in CUMS group (Fig. 4B, *P*<0.05). Compared with the CUMS group, CUMS + EGCG group rats showed a significant decrease in the number of TUNEL-positive cells in the CA1 region (Fig. 4B, *P*<0.05). These results further indicated that EGCG decreased CUMS-induced apoptosis in CA1 regions of rats. While CUMS +EGCG + CQ group rats had higher percentage of TUNEL-positive cells as compared with that of CUMS + EGCG group rats (Fig. 4B, *P*<0.05), suggesting that the protective effects of EGCG treatment could be abrogated by CQ in CUMS rats.

### EGCG rescues CUMS-induced autophagic flux impairment in the CA1 region

Since neurons are under strict surveillance rendering them subject to autophagy at any time, it is possible that autophagy may be involved in the injury of neurons in CUMS rats. Therefore, we performed TEM and western blotting studies to evaluate the effects of EGCG on autophagic flux induced by CUMS.

**Figure 5:**
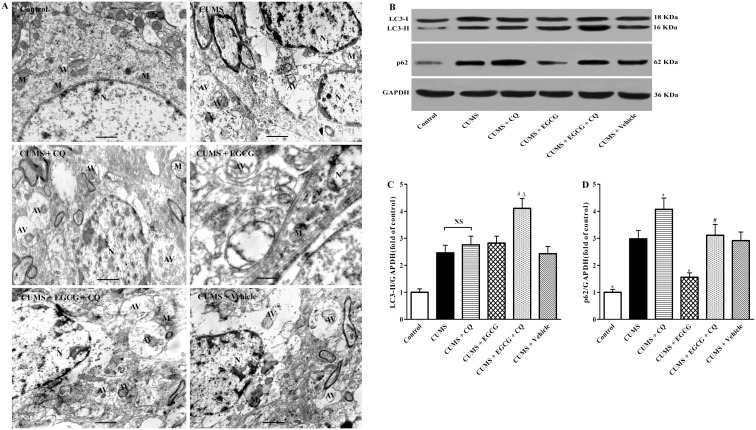
EGCG effects on CUMS–induced changes in ultra-structure and autophagic flux in CA1. TEM and western blotting were conducted to evaluate autophagic flux. (A) TEM was used to examine autophagosome formation. Control group shows ultra-structure of neuronal cells in the CA1 hippocampus under control conditions, characterized by normal structure of the mitochondria (M) and the nucleus (N) with evenly distributed chromatin. CUMS group, CUMS + CQ, CUMS + EGCG + CQ and CUMS + vehicle group show ultra-structurally changed neuronal cells in the CA1 sector of the hippocampus, including abnormal mitochondria (M), heterogeneous lysosomes, and increased autophagic vacuoles (AVs)-containing impaired mitochondria (M) and unrecognized aggregate of electron-dense material compared with the control. CUMS + EGCG group indicates that EGCG-treatment markedly attenuates CUMS-induced ultra-structure impairment in CA1, characterized by decreased swollen mitochondria, heterogeneous lysosomes, and dysfunctional lysosomes-containing dense granules compared with those in the CUMS group rats. The presented photos are representative of the three animals used in each experimental group. (B) Western blotting results showing the effects of EGCG on LC3-I (18 kDa), LC3-II (16 kDa), and p62 (62 kDa) protein levels in CA1. Each lane contained 30 µg proteins for all experiments. (C), and (D), Densitometry analysis of LC3-II and p62 protein levels was performed using three independent experiments. GAPDH was used as control for protein loading. Control, control group; CUMS, chronic unpredictable mild stress group; CUMS + CQ, CQ administration followed by CUMS group; CUMS + EGCG, EGCG treatment followed by CUMS group; CUMS + EGCG + CQ, co-administration with EGCG and CQ followed by CUMS group; Vehicle, vehicle treatment followed by CUMS group. The drugs (EGCG and CQ) were given to rats 30 min before the stress exposure. Data are shown as mean ± SEM (n = 3 for each group), and one-way ANOVA with Newman–Keuls post hoc analysis was used. NS means no significant difference. *P<0.05 versus CUMS group, ^#^ *P*<0.05 versus CUMS + EGCG group, ^Δ^ *P*<0.05 versus CUMS + CQ group. Scale bar, 500 m.

First, we examined whether CUMS impaired autophagic flux process. As shown in Fig. 5A, TEM results showed significant morphologic changes in the CA1 hippocampus of CUMS group rats, characterized by increased number of mitochondrial swelling and autophagic vacuoles (AVs). These changes suggested that CUMS induced autophagy stress. To further confirm whether up-regulated or impaired autophagic degradation pathway contributed to the increased AVs in CA1 regions of CUMS rats. The expression of two critical markers in the process of autophagic flux, LC3-II, and p62 levels were determined by western blotting (Fig. 5B). Comparison of LC3-II western blots of CA1 extracts of control group with CUMS group rats revealed a significant increase in LC3-II levels in CUMS group rats (Fig. 5C, *P*<0.05). In this study, we further examined whether CUMS impaired autophagic flux by comparing accumulation of LC3-II with and without inhibition of lysosomal degradation. We blocked lysosomal degradation by CQ. As shown in Fig. 5C, accumulation of LC3-II was markedly increased in CUMS group as compared with that in control group. However, LC3-II level in CUMS + CQ group was not further increased as compared with CUMS group, indicating that autophagic flux was impaired in CUMS group. It is well known that the ubiquitin-binding protein p62/SQSTM1 is an autophagy substrate, which upon direct binding to LC3-II incorporates into autophagosomes and is efficiently degraded by autophagy. Thus, when autophagic degradation pathway is blocked, p62 accumulation occurs. To further assess the hypothesis that LC3-II increase in CUMS group rats was a consequence of defective autophagic flux rather than increased autophagic activity, we evaluated p62 levels by western blotting in the CA1 of rats. Western blotting analysis showed that p62 levels of CUMS group rats were much higher than those of control ones (*P*<0.05). These data suggested a disruption of the autophagic flux in CA1 regions of CUMS rats.

To confirm whether EGCG exerted its neuroprotection via the regulation of the autophagic flux, we examined the effect of EGCG (25 mg/kg) on morphological–ultrastructural alterations and levels of LC3-II and p62. Our data showed that the number of autophagosomes notably reduced, concomitantly with significantly decreased swollen mitochondria in CA1 regions of EGCG group rats as compared with that of CUMS group (Fig. 5A). Western blotting results indicated that CUMS group rats treatment with EGCG did not further enhanced the accumulation of LC3-II, but LC3-II level in CUMS + EGCG + CQ group was dramatically increased as compared with that in CUMS + EGCG group (*P*<0.05). Furthermore, EGCG treatment markedly decreased the expression of p62 in CA1 (*P*<0.05), as compared with CUMS group rats. These results demonstrated that EGCG could restore autophagic flux in CA1 region impaired by CUMS.

### EGCG decreases Aβ_1−42_ levels in CA1 regions of CUMS rats

**Figure 6:**
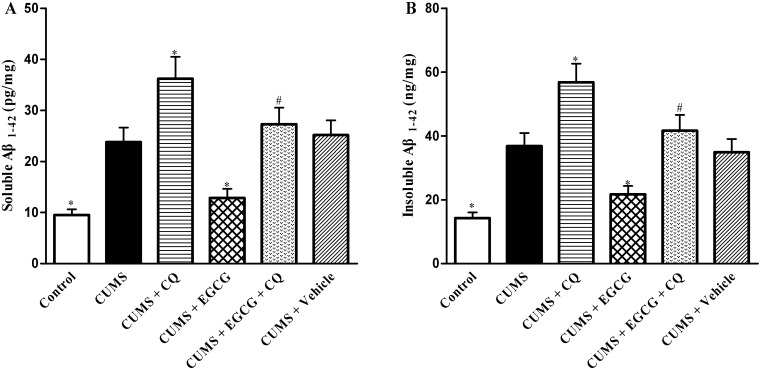
EGCG treatment reduces both soluble and insoluble Aβ_1–42_ levels in the hippocampal CA1 regions of CUMS rats. (A) The soluble Aβ_1–42_ levels in different groups were detected by ELISA. Data were presented as (pg/mg protein). (B) Insoluble Aβ_1–42_ levels in different groups were measured by ELISA. Data were presented as (ng/mg protein). Control, control group; CUMS, chronic unpredictable mild stress group; CUMS + CQ, CQ administration followed by CUMS group; CUMS + EGCG, EGCG treatment followed by CUMS group; CUMS + EGCG + CQ, co-administration with EGCG and CQ followed by CUMS group; Vehicle, vehicle treatment followed by CUMS group. The drugs (EGCG and CQ) were given to rats 30 min before the stress exposure. Data are shown as mean ± SEM (n = 4 for each group). *P<0.05 versus CUMS group, ^#^ *P*<0.05 versus CUMS + EGCG group.

Excessive accumulation of Aβ peptide in the brain is the key pathological change in AD-like cognitive dysfunction. Considering the crucial role of autophagy in Aβ_1–42_ production, we determined the effect of EGCG on the accumulation of Aβ_1–42_ in the hippocampal CA1 region by ELISA analysis. As shown in Fig. 6, the soluble (Fig. 6A) and insoluble Aβ1-42 (Fig. 6B) levels in the CA1 regions of the different groups were measured by ELISA, respectively. The results showed that both soluble and insoluble Aβ_1–42_ contents in the CA1 of CUMS group rats were significantly higher than those of control ones (*P*<0.05, Fig. 6A, B). Aβ_1–42_ levels in the CA1 of CUMS + CQ group were much higher than those in CUMS group (*P*<0.05, Fig. 6A, B). As expected, treatment of EGCG obviously decreased such a high level of Aβ_1–42_ in the CA1 areas of CUMS (*P*<0.05, Fig. 6A, B), while CQ could reverse the EGCG-induced Aβ_1–42_ reduction in CA1 regions of CUMS rats (*P*<0.05, Fig. 6A, B). This result thereby suggested that EGCG decreased Aβ_1–42_ levels and via restoring autophagic flux.

### mTOR/p70S6K pathway is involved in EGCG-regulated autophagy in CUMS rats

**Figure 7:**
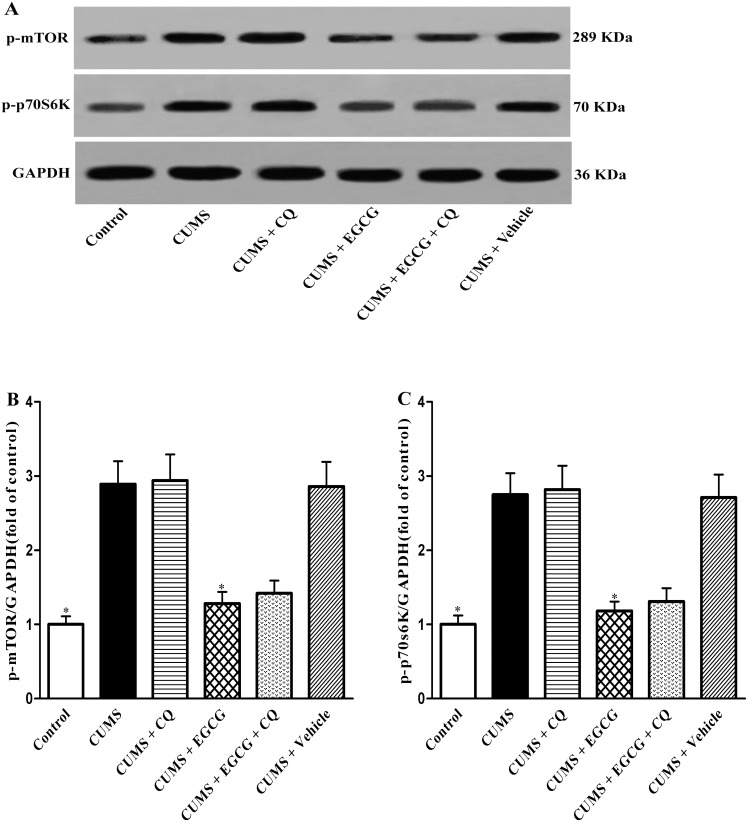
EGCG inhibits CUMS–induced activation of the mTOR pathway in CA1. (A) The effects of EGCG on p-mTOR (289 kDa) and p-p70S6K (70 kDa) protein levels in CA1 were investigated by western blot analysis. Each lane contained 30 µg proteins for all experiments. (C, D) Densitometry analysis of p-mTOR and p-p70S6K protein levels was performed using three independent experiments, respectively. GAPDH was used as control for protein loading. Control, control group; CUMS, chronic unpredictable mild stress group; CUMS + CQ, CQ administration followed by CUMS group; CUMS + EGCG, EGCG treatment followed by CUMS group; CUMS + EGCG + CQ, co-administration with EGCG and CQ followed by CUMS group; Vehicle, vehicle treatment followed by CUMS group. The drugs (EGCG and CQ) were given to rats 30 min before the stress exposure. Data are shown as mean ± SEM (n = 3 for each group). *P<0.05 versus CUMS group.

The mTOR signaling pathway is known to regulate autophagy, and inhibition of mTOR results in the activation of autophagy. P70S6 kinase (p70S6K) is the substrate of mTOR, and its phosphorylation (p-p70S6K) reflects the activity of mTOR. Considering the achieved results of EGCG-restored autophagic flux in CUMS rats, we next explored the mechanism underlying such effects by examining the potential inhibition of the mTOR-mediated pathway using western blot assay. As shown in Fig. 7, the levels of p-mTOR (Fig. 7A and 7B) and p-p70S6K (Fig. 7A and 7C) in CUMS group rats were significantly higher than those in the control group (both *P*<0.05), indicating the high mTOR activity in CUMS rats. As expected, p-mTOR (Fig. 7A and 7B) and p-p70S6K (Fig. 7A and 7C) levels in CUMS + EGCG group were significantly lowered as compared with those in CUMS group, respectively (both *P*<0.05). These data suggest that the mTOR pathway was involved in EGCG-regulated autophagic flux.

## Discussion

In the present study, we applied a paradigm of CUMS rat model, which shows exacerbating memory impairment, to investigate the effects of stress on Aβ_1–42_ accumulation and autophagy in CA1 and how EGCG attenuates CUMS-induced learning and memory impairment. Our major findings include: (1) CUMS resulted in increased intracellular Aβ_1−42_ accumulation, cells loss, and autophagic flux impairment in CA1 of rats; (2) EGCG-treatment efficiently ameliorated learning and memory deficits, and autophagic flux impairment induced by CUMS; (3) EGCG rescue of the autophagic influx may be through inhibition of mTOR signaling pathway. These results suggested that EGCG may be exploited as a potential therapeutic reagent for the treatment of learning and memory deficits associated with abnormal autophagy.

It has been reported that chronic stress can exacerbate neuro-degeneration and impair cognitive performance in AD transgenic mice [2]. Our results demonstrated that CUMS markedly aggravated learning and memory impairment and also increased cell loss and Aβ_1–42_ accumulation in CA1. These findings are consistent with those analogous results obtained in AD transgenic mice after similar paradigms of chronic stress [2]. In the present study, CUMS model rats exhibited elevated glucocorticoid levels, one of characteristics of chronic stress. In previous studies [22], [25], [30], the effects of EGCG on AD memory deficits have been investigated in several animal models. In this study, our results showed that 15-week-old male rats exposed to 4-week CUMS demonstrated a remarkable deficiency in the hippocampus-dependent tasks of MWM. Furthermore, we found that EGCG treatment for 4 weeks rescued spatial learning and memory deficits as indicated by reduced escape latency as well as increased target quadrant occupation in these CUMS rats. As shown in Fig. 1, EGCG treatment had no significant effect on the plasma glucocorticoid levels of CUMS rats, suggesting that EGCG improvement of CUMS-induced memory deficits was independent of glucocorticoid levels. Interestingly, this protective effect of EGCG can be antagonized by CQ, an autophagic flux inhibitor. These findings suggested that EGCG improvement of CUMS-induced memory deficits may be associated with the complete autophagic flux.

Recent studies have suggested that autophagy impairment is involved in cognitive dysfunction, such as AD [11], [17], [31]. We have provided evidence for dysregulated autophagy in CA1 underlying the effects of CUMS on neurodegeneration in the hippocampus of CA1. Specifically, our electron microscopic analysis revealed that CUMS resulted in a significant increase in the number of AVs. However, the accumulation of AVs is not always indicative of autophagy induction, as it may represent either an increase in generation of AVs and/or a block in autophagosomal maturation and/or the completion of the autophagic flux. Therefore, western blotting method was employed to evaluate the expression of proteins related to autophagic flux in CA1, including LC3-I, LC3-II, and p62 protein levels. We found LC3-II level was markedly increased in CUMS group. Interestingly, LC3-II level in CUMS + CQ group was not significantly increased as compared with that in CUMS group. Furthermore, LC3-II accumulation (Fig. 5C) was accompanied by a drastic elevation of p62 level (Fig. 5D) in CA1 of CUMS group as compared with the control one. These changes were also accompanied by increased number of autophagic vacuoles as noted by TEM (Fig. 5A). Taken together, these data strongly supported the hypothesis of a blockade in the autophagic flux in CUMS rats.

In this study, we also found a significant increase in Aβ_1–42_ accumulation in CA1 of CUMS rats. Two possible explanations are proposed. First, Aβ_1–42_ accumulation may be due to the increased glucocorticoid level that reportedly aggregates Aβ_1–42_ [2]. It is also possible due to the CUMS-reduced degradation of Aβ_1–42_ [1]. Several degradation pathways of Aβ_1–42_ have been proposed, including ubiquitin-dependent proteasome pathway and autophagy-lysosomal pathway [32], [33]. Our results showed that a significant increase in Aβ_1–42_ accumulation in CA1 of CUMS rats was associated with elevated LC3-II and p62 levels. These data suggested that CUMS-induced autophagic flux impairment decreased the clearance of autophagic substrates, likely resulting in Aβ_1–42_ accumulation in CA1.

Among the numerous components involved in the regulation of autophagy and neurodegenerative diseases, mTOR signaling is a key component that coordinately regulates the balance between neurodegeneration and autophagy in response to stress [16], [34]. To further explain the mechanisms for the autophagic flux impairment induced by CUMS, we examined the phosphorylation levels of mTOR and its downstream effector p70S6K in CA1. The changes in their phosphorylation levels represent as the alternations of mTOR activities [35]. Our results showing that CUMS resulted in a significant increase in the phosphorylation levels of mTOR and p70S6K strongly suggest that mTOR signaling plays a critical role in autophagic flux impairment induced by CUMS.

It has been established that impairment of autophagy contributes much to the abnormal protein accumulation (e.g., Aβ_1–42_) in several age-dependent neurodegenerative diseases, including AD [17], [18] and Parkinson’s disease [36]. Compounds with activity of autophagy regulation have been identified to delay the AD pathology process, and inhibition of mTOR could increase Aβ_1–42_ clearance and rescue memory impairment in AD model mice via enhancing autophagy [27], [37]. EGCG has been shown to exert neuroprotection. Several studies have suggested that autophagy is one of the therapeutic targets for EGCG [26], [27], [38]. In the present study, the effects of EGCG on CUMS-induced autophagic influx impairment, Aβ_1–42_ accumulation, and pathological changes have been established. Our results showed that EGCG treatment significantly reduced the number of AVs, and the expressions of p62 protein in CA1 of CUMS group rats. Meanwhile, EGCG treatment elevated the LC3-II level in CUMS rats. We also observed that EGCG treatment decreased Aβ_1–42_ accumulation and neuron loss in CA1 region of the CUMS rats, which is in agreement with previous observations in transgenic mice [1]. Activation of autophagy has protective effects in mouse and fly models of cognitive dysfunction. Therefore, it is not surprising that restoration of autophagic activity by EGCG decreased the number of Aβ_1–42_ aggregates and improved cell survival. However, CQ, a specific inhibitor of autophagy-lysosomal pathway, could completely abolish these effects of EGCG. These data have therefore suggested that EGCG protection of rats from CUMS-induced learning and memory impairment is likely associated with restoring autophagic flux.

Numerous studies have demonstrated that can regulate mTOR signaling in many cell lines, for instance, Huang et al. [39] have reported that EGCG inhibited the mTOR pathway through AMPK activation in cancer cells, Chen et al. [40] found that EGCG inhibited the proliferation of cancer stem cells via down-regulation of mTOR pathway, Peairs A et al. [41] reported that EGCG attenuated inflammation in MRL/lpr mouse mesangial cells via the PI3K/Akt/mTOR pathway. In this study, we have revealed that EGCG rescue of autophagic influx is likely dependent of the inhibition of the mTOR pathway. After 4-week EGCG treatment, both p-mTOR and p-p70s6K were significantly reduced in CA1 regions of CUMS rats (Fig. 7). Since inhibition of the mTOR signaling is well known to activate autophagy [34], our data suggest that EGCG may rescue autophagic flux through the inhibition of mTOR pathway.

In summary, this study has demonstrated that EGCG could improve CMUS-induced memory impairment in rats. The mechanism underlying the protective effect of EGCG may be associated with rescuing CUMS-impaired autophagic flux through the mTOR pathway. EGCG treatment restored autophagic flux, a key step for autophagic degradation of Aβ_1–42_, which may help reduce the accumulation of Aβ_1–42_ and protects cells against CUMS-induced injury in CA1 regions. These data shed new light on a novel mechanism underlying EGCG amelioration of CUMS-induced memory deficits in rats.
