# Correction: Epigallocatechin-3-Gallate Attenuates Impairment of Learning and Memory in Chronic Unpredictable Mild Stress-Treated Rats by Restoring Hippocampal Autophagic Flux

**DOI:** 10.1371/journal.pone.0117649

**Published:** 2015-02-06

**Authors:** The PLOS ONE Staff

The last author’s affiliation is incorrect. Duan-Fang Liao should be affiliated with #2 Division of Stem Cell Regulation and Application, State Key Laboratory of Chinese Medicine Powder and Medicine Innovation in Hunan, Hunan University of Chinese Medicine, Changsha, People’s Republic of China
